# Developer Perspectives on Potential Harms of Machine Learning Predictive Analytics in Health Care: Qualitative Analysis

**DOI:** 10.2196/47609

**Published:** 2023-11-16

**Authors:** Ariadne A Nichol, Pamela L Sankar, Meghan C Halley, Carole A Federico, Mildred K Cho

**Affiliations:** 1 Center for Biomedical Ethics Stanford University School of Medicine Stanford, CA United States; 2 Department of Medical Ethics & Health Policy University of Pennsylvania Philadelphia, PA United States

**Keywords:** machine learning, ML, algorithms, health care quality, responsibility, ethics, machine learning predictive analytics, MLPA, developers

## Abstract

**Background:**

Machine learning predictive analytics (MLPA) is increasingly used in health care to reduce costs and improve efficacy; it also has the potential to harm patients and trust in health care. Academic and regulatory leaders have proposed a variety of principles and guidelines to address the challenges of evaluating the safety of machine learning–based software in the health care context, but accepted practices do not yet exist. However, there appears to be a shift toward process-based regulatory paradigms that rely heavily on self-regulation. At the same time, little research has examined the perspectives about the harms of MLPA developers themselves, whose role will be essential in overcoming the “principles-to-practice” gap.

**Objective:**

The objective of this study was to understand how MLPA developers of health care products perceived the potential harms of those products and their responses to recognized harms.

**Methods:**

We interviewed 40 individuals who were developing MLPA tools for health care at 15 US-based organizations, including data scientists, software engineers, and those with mid- and high-level management roles. These 15 organizations were selected to represent a range of organizational types and sizes from the 106 that we previously identified. We asked developers about their perspectives on the potential harms of their work, factors that influence these harms, and their role in mitigation. We used standard qualitative analysis of transcribed interviews to identify themes in the data.

**Results:**

We found that MLPA developers recognized a range of potential harms of MLPA to individuals, social groups, and the health care system, such as issues of privacy, bias, and system disruption. They also identified drivers of these harms related to the characteristics of machine learning and specific to the health care and commercial contexts in which the products are developed. MLPA developers also described strategies to respond to these drivers and potentially mitigate the harms. Opportunities included balancing algorithm performance goals with potential harms, emphasizing iterative integration of health care expertise, and fostering shared company values. However, their recognition of their own responsibility to address potential harms varied widely.

**Conclusions:**

Even though MLPA developers recognized that their products can harm patients, public, and even health systems, robust procedures to assess the potential for harms and the need for mitigation do not exist. Our findings suggest that, to the extent that new oversight paradigms rely on self-regulation, they will face serious challenges if harms are driven by features that developers consider inescapable in health care and business environments. Furthermore, effective self-regulation will require MLPA developers to accept responsibility for safety and efficacy and know how to act accordingly. Our results suggest that, at the very least, substantial education will be necessary to fill the “principles-to-practice” gap.

## Introduction

Machine learning predictive analytics (MLPA) applications have attracted significant investment over the past few years and are increasingly used in the health care industry [1,2]. Distinct from machine learning (ML) used in medical devices for diagnostic purposes, some of these tools specifically aim to improve health care efficiency and curb burgeoning costs [3]. For example, an MLPA tool was recently developed using electronic health record data from thousands of patients, with the goal of improving quality and reducing the costs of intensive care unit care [4]. Other examples include MLPA applications developed for predicting physiological deterioration [5], hospital readmissions [6], and disease forecasting [7]. MLPA applications have the potential to improve individual patient and population health and to reduce health care costs. However, MLPA applications also have the potential to harm patients and patient trust in health care through systematic error, violations of privacy, lack of transparency, and exacerbation of health disparities [8-12]. Commentary recognizes the potential benefit of health care MLPA but demands that its deployment come with guardrails to limit its risks [8-12]. In response, scholarly efforts have focused on codes of ethics, factors supporting their adherence [13,14], and on technical remedies [15,16], as well as on the need to understand more about how ML developers understand their work [17].

Demands to rein in or closely monitor systems relying on algorithmically driven predictions cut across many fields including finance, government, marketing, and medicine [18]. Concerns about MLPA for health care began to garner interest within computer science in the mid-2010s [19,20] and captured public attention a few years later with the publication of studies demonstrating racial bias in algorithms used in criminal justice systems, health care services, and credit agencies [10,21-23]. A steady stream of proposals to better manage artificial intelligence (AI) tools for social benefit has continued since, with a recent publication identifying 200 studies from the past decade offering guidelines to oversee the technology’s development [24]. The volume of publications evinces the persistence of social concern about potential harms resulting from the rapid spread of AI tools but also the challenge their management poses. Heightened concerns are voiced about health care MLPA over other fields because of its potential direct and broad effect on human health and well-being [25].

A few features stand out in these discussions. First, an algorithm’s mechanism or logic can be opaque. The process that produces a particular result can be indiscernible even to the person who developed the algorithm. Second, algorithms are iterative, meaning they are designed to continually process data, update parameters, and produce new results. Moreover, the pipeline through which algorithms are produced entails many steps and many types of expertise. The work often takes place across several high-pressure settings. As an emergent, complex, and rapidly evolving technology produced through a distributed, multistep process, distinct for its opacity and its disposition to continuously update, MLPA challenges basic oversight.

Academic and regulatory leaders have proposed a variety of approaches to confronting these challenges within health care. For example, the Food and Drug Administration (FDA) in the United States has proposed moving away from its long-standing product-based assessments and toward a new process-based form of regulation when handling software-based medical devices, or software as a medical device (SaMD) [26]. By focusing on the process by which an ML health care tool is created, rather than the product or tool itself, the FDA circumvented at least some of the problems with overseeing MLPA, such as the need to treat an updated algorithm as a new product requiring review and approval. The approach, however, addresses only some MLPA-related challenges, as only a relatively small subset of MLPA tools qualify as SaMD and are thus subject to FDA regulation [27,28]. Further, the FDA’s process-based approach itself remains nascent, contentious [29], incompletely authorized [30], and in need of additional piloting [31]. Outside of the formal regulatory environment, additional efforts to fashion guardrails for MLPA that facilitate the technology’s safe deployment in health care include research to devise technical fixes to improve and standardize methods to detect sources of bias [32-34] and to promote “explainable AI” that increases algorithmic transparency [15]. These are promising but still imperfect solutions: observers report that some of these proposals may be difficult to enact [16], and others may not be “fully operable in practice” [35].

Exploring developer perspectives on their roles and responsibilities for mitigating the potential harms of MLPA can contribute to building a broad knowledge base on which to construct practices supporting effective and responsible MLPA. However, relatively little research has addressed this specific question. To contribute to this effort, we conducted interviews with MLPA developers designed to capture their perspectives on the potential harms of their work, factors that influence these harms, and their role in mitigation.

## Methods

### Recruitment

From July 2019 to July 2020, we recruited individuals who were working for US-based organizations involved in developing MLPA tools for use in health care settings. We selected individual organizations based on our previously published analysis of the landscape of predictive analytics in health care [36], which included a range of organizational types and sizes. The eligibility criteria for study participation was employment at 1 of the US-based organizations identified as developing MLPA tools for health care use. We identified the organizations included in the landscape analysis by first assessing 4 databases—LexisNexis, PubMed, Web of Knowledge, and Indeed.com—using search terms such as “hospitals,” “health care organizations,” “machine learning,” and “predictive analytics”; using set inclusion and exclusion criteria to determine relevant products; and then performing data extraction on the organization’s product website content to classify them. The sample consisted of computer software and IT companies, including those specifically focused on health care, as well as health insurers and hospital systems. In addition, we classified organizations by size based on the number of employees (small=1-50, medium=51-1000, and large=>1000), as specified in the LinkedIn page for each organization. All organizations that were identified by our initial landscape analysis of 4 databases (LexisNexis, PubMed, Web of Knowledge, and Indeed.com) had LinkedIn pages. Organizations were classified by type and size in order to provide a clear picture of the organizations included in the sample. Of the 96 organizations identified, we used quota sampling [37] to ensure the diversity of perspectives, selecting 15 that were representative of the range of organizations, both in terms of type and size. Since this was a qualitative study, the goal was to capture the fullest range possible of organizations according to qualitative characteristics such as the type of organization (eg, software company vs hospital), rather than to achieve quantitative representation.

From these organizations, we initially identified potential participants through LinkedIn, the most widely used professional networking platform in the United States [38], reviewing search results by organization for keywords such as data scientist, software engineer, or manager. We contacted individuals to participate through LinkedIn’s direct messaging feature. To minimize bias in selection, individuals were chosen for contact based on the order with which they appeared in each organization’s employee search, filtering by relevant keywords such as data scientist, software engineer, or manager. To identify additional participants who might not be represented on LinkedIn, we also used a snowball sampling approach. To examine the MLPA development process from different perspectives, we intentionally included participants representing a variety of roles, including data scientists, software engineers, project managers, and executive leaders, among others.

### Data Collection

Each participant completed a 1-hour semistructured interview through videoconference. The average recorded interview time duration was 49 minutes. We iteratively developed the interview guide through pilot interviews with current MLPA developers. The interview guide (Multimedia Appendix 1) included questions on the participants’ background and training, company and MLPA product goals in health care, facilitators and barriers to product development, and potential benefits and harms of these products.

### Ethical Considerations

Our study was approved by the institutional review board of Stanford University (protocol 48902, FWA00000935). Participants were informed that they could opt out at any time during the interview process. The interview data were deidentified. All participants received an electronic gift card of US $100 for participation.

### Data Analysis

Interviews were audio recorded, transcribed verbatim, and deidentified. We analyzed the data using the mixed methods analytic software Dedoose (version 8.3; SocioCultural Research Consultants) [39]. All team members reviewed subsets of the interview transcripts and then identified and discussed the most prevalent themes seen across interviews with the whole team. Based on those discussions, a list of concepts was generated as an initial codebook. The team then iteratively refined the codebook through multiple rounds of provisional coding. Once the codebook was finalized, at least 2 team members independently coded each interview, resolving any coding differences through team consensus. To further examine participant perceptions of the potential harms of MLPA in health care and their attitudes toward handling those harms, we then reviewed all data coded to ideas associated with mitigating harms across all participants to identify consistency and variability in narratives based on individual and organizational characteristics.

## Results

### Participant Characteristics

We selected 15 organizations from 96 originally identified, on the basis of organizational characteristics. We made this selection in a way to ensure that all of the organizational characteristics—company sizes, types, and product types—were represented in similar proportions to the larger sample (Table 1).

**Table 1:** Organizational characteristics.

Organization characteristics			Organizations (n=15), n (%)
**Size**			
	Small (1-50 employees)	6 (40)	
	Medium (51-1000 employees)	3 (20)	
	Large (>1000 employees)	6 (40)	
**Organization type**			
	Computer software or IT—health care	10 (67)	
	Computer software or IT—general	2 (13)	
	Health insurer	2 (13)	
	Provider (hospital or health system)	1 (7)	
**Product types**			
	Disease onset and progression	11 (73)	
	Treatment	7 (47)	
	Cost and utilization	9 (60)	
	Decompensation and adverse events	4 (27)	
	Admissions and readmissions	5 (33)	

Of the 76 prospective participants contacted, 40 (53%) agreed to participate. The majority (29/40, 72%) of participants worked at health care–oriented computer software and IT companies. Almost two-thirds (25/40, 62%) of participants held roles that involved both working directly with data in MLPA development and other functions, such as leadership. A total of 40% (16/40) participants occupied high-level management roles. A total of 35% (14/40) held health-related advanced degrees. Participant and company characteristics are provided in Table 2, and individual-level data are provided in Multimedia Appendix 2.

**Table 2:** Participants’ professional and academic characteristics.

Participant characteristics		Participants (n=40), n (%)
**Management levels^a^**		
	None	15 (38)
	Midlevel	9 (22)
	High level	16 (40)
**Data interaction levels^b^**		
	Data only	15 (38)
	Data+	25 (62)
**Academic backgrounds**		
	Bachelor’s degree	11 (28)
	Health-related master’s degree	5 (12)
	Non–health-related master’s degree	6 (15)
	Health-related PhD^c^	5 (12)
	Non–health-related PhD	9 (22)
	Medical degree	4 (10)
**Type of organization**		
	Computer software and IT—health care	29 (72)
	Computer software and IT—general	3 (8)
	Health insurer	3 (8)
	Hospital	5 (12)
**Number of employees at organization**		
	1-50	19 (48)
	51-1000	5 (12)
	>1000	16 (40)

^a^None refers to participants without managerial duties; mid-level refers to participants with some managerial duties; and high-level refers to participants with extensive managerial duties.

^b^Data only refers to participants who handle and work directly with the data in their daily work; Data+ refers to participants who not only work with data but also perform other functions within their organization.

^c^PhD: Doctor of Philosophy.

### Developer Perspectives: Potential Harms, Drivers of Harms, and Responses

#### Overview

**Figure 1:**
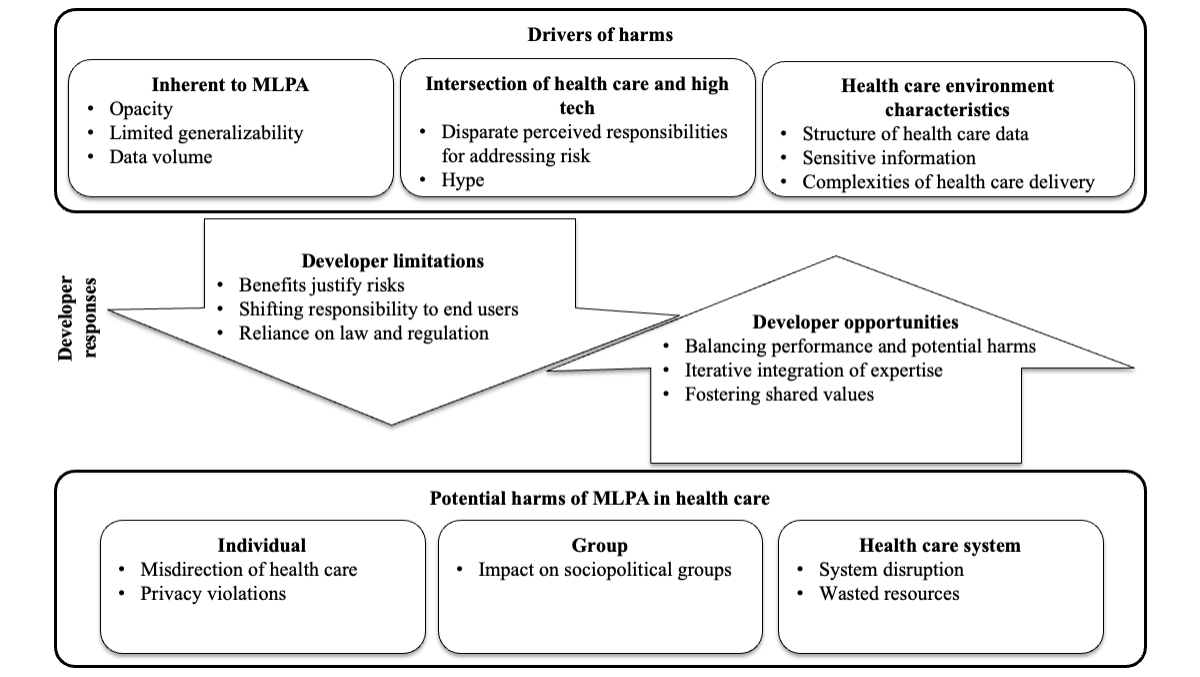
Developer perspectives on harms, drivers, and responses. MLPA: machine learning predictive analytics.

In response to interview questions about the process and challenges of producing health care MLPA, developers named a range of potential harms associated with the technology. They also speculated about factors that might exacerbate or drive MLPA-associated harms, as well as about opportunities to respond to these drivers and perceived limitations of their own responsibilities to do so. Although developers varied in the extent to which they were able to articulate harms, drivers, and responses, we were unable to identify individual or organizational characteristics that appeared to be associated with a greater appreciation of these issues overall. Below we first present an analysis of developers’ comments about potential harms, followed by an analysis of drivers of harms, and then an analysis of comments about possible responses to drivers of potential harms. We end with a description of developer perspectives on responsibility for responding to these potential harms. Figure 1 provides a framework illustrating the domains developers identified related to drivers of harms, potential developer responses, as well as potential harms as articulated by participants. Those developer responses that represent opportunities to mitigate against potential harms are indicated by the upward arrow, while those that represent limitations that may facilitate (or fail to prevent) drivers leading to harms are illustrated by the downward arrow. The boxes surrounding the categories of “Drivers of Harms” and “Potential Harms of MLPA in Healthcare” indicate that the relationships depicted are between each of these domains as a whole and do not suggest one-to-one relationships between individual drivers and harms. While certain drivers may indeed be directly related to certain developer responses and potential harms, our interviews were not designed to ascertain this level of mapping of relationships.

#### Potential Harms of MLPA in Health Care

Participants identified 3 categories of potential harms from the use of MLPA in health care, including harms to individuals, vulnerable sociopolitical groups, and the health care system more broadly. Multimedia Appendix 3 provides the longer passages from which quotes cited here are excerpted.

Developers identified 2 types of harms to individuals: privacy violations and what we have labeled “misdirection of health care.” The latter refers to instances when MLPA tools might misdirect health care resources or interventions either away from those who needed more care or toward those who did not. As participants P02 and P24 explained, a patient could potentially not receive treatment if a model inappropriately identified them as low risk based on how developers designed the tool. Developers’ concerns regarding violation of privacy focused on the sharing of sensitive health information not germane to a developer’s task (participant P30).

The primary harm to groups mentioned by developers was the possibility of systematic bias in an algorithm’s outcomes leading to medically unjustified differences in treatments among certain sociopolitical groups. Developers such as participant P06 explained this potential harm by referencing a recent, high-profile study that demonstrated systematic bias in a widely used MLPA tool that resulted in the allocation of health care resources away from African American patients [10]. Developers also expressed concern that optimizing specifically for health care costs in MLPA-based models could lead to biased algorithms and subsequent harm to already vulnerable sociopolitical groups (participant P17).

Developers identified 2 main potential harms to the health care system: system disruption and wasted resources. Forms of potential health care system disruption included atrophy of physicians’ skills and alarm fatigue among health care providers (eg, participants P19 and P08), as well as the potential for unmonitored MLPA to cause system disruption by contradicting existing health care and public health praxis (eg, participant P22). Developers raised concerns about wasted resources due to the magnitude of time and money being directed toward the development of MLPA tools for health care. They voiced concern that emphasis on MLPA tools, many of which ultimately may not prove to be useful, could drive resources away from other potential interventions for improving health care (eg, participants P16 and P22).

### Drivers of Potential Harms of MLPA in Health Care

#### Overview

We defined drivers of harms as conditions or factors that participants identified as contributing to the likelihood or magnitude of potential harms associated with MLPA. Drivers fell into three broad categories: (1) characteristics that developers consider inherent in MLPA; (2) characteristics of the health care environment in which developers were working; and (3) factors generated by the intersection of health care and the high-tech industry. Multimedia Appendix 4 provides the longer passages from which quotes cited here are excerpted.

#### Factors Developers Characterized as Inherent in MLPA Technology

Drivers that participants characterized as inherent in MLPA technology, or as inevitable consequences of its creation and operation, included opacity, limited generalizability, and data volume. Opacity referred to the limited extent to which observers can understand how an MLPA tool generates its predictions. For example, participant P18 commented that “the scariest thing about machine learning in general, is if you have a model that’s not really explainable and it’s pretty predictive and you don’t know why it’s predictive.” Participants also highlighted that racial bias could operate in an MLPA tool, hidden from view, due to opacity (participant P35).

Statements also highlighted possible harms resulting from unrecognized limits to generalizability; for example, developers noted that failing to account for the distinctiveness of populations or settings in which algorithms were trained or tested could result in MLPA that might not readily translate to heterogenous, real-world patient populations or settings (participants P19 and P08).

Participant concerns regarding data volume focused on MLPA’s requirement for vast amounts of data. Powerful as large data sets might be, participant P03 explained, large amounts of data do not necessarily mean better results, as much as an opportunity to make bigger mistakes. Furthermore, preoccupation with the volume of data that MLPA is capable of analyzing may lead developers, and particularly those with backgrounds outside of health care, to value volume over quality, based on the assumption that “the more data the better” (participant P20).

#### Health Care Environment

Participants also identified drivers of harms related to the characteristics of the health care environment in which MLPA was being developed, including the structure of health care data, the sensitive nature of these data, and the complexity of health care delivery.

Features related to the structure of health care data included the multiple formats in which data are entered into electronic health records (participant P42); data storage in multiple, stand-alone sources, such as those for prescriptions or lab results; and the lack of standardization governing what information is submitted or available (participant P24). As participant P42 highlighted, the complex structure of health care data made it harder for developers to recognize possible problems with their algorithms and, as a result, “you don’t realize that you’ve just overfitted a model to a big pile of garbage.”

Participants also recognized the sensitive nature of the health care data needed to train MLPA models as a driver of harm because these data demanded careful handling (participant P05). Participant P18 noted as well that the volume of data demanded by MLPA increased the probability that a person could be reidentified, even from anonymized data.

Developers also recognized that the complexity of health care delivery more broadly hampered their ability to recognize important nuances in data sets, leading to wasted efforts or errors in prediction (participant P33). Participant P05 explained that, when attempting to understand complex treatment regimens, for example, developers could quickly get themselves “into very murky waters.”

#### Intersection of Health Care and the High-Tech Industry

Developers identified drivers of potential harms specific to the intersection of the powerful high-tech industry that drives MLPA development and the equally powerful but substantively different health care industry, including, for example, a disparate sense of responsibility for addressing risk in health care versus the high-tech industry and, in the latter, a greater acceptance of overly optimistic expectations, or “hype.”

Comments about the differences in perceived responsibility between high tech and health care contrasted high tech’s embrace of “failing forward,” a popular premise in high tech that characterizes mistakes as a natural and financially expedient part of innovation [40] with medicine’s commitment to prioritize caution and patient safety, while pursuing its goals of relieving pain and promoting health. As participant P03 noted, there is a “tolerance to failure” in tech, which is “antithetical to medicine.” They note that, in medicine, “you’re not allowed to fail with people.” Participants also suggested that the settings in which MLPA originally evolved, such as marketing and finance, might contribute to the disparate sense of responsibility for addressing risk because in those settings developers’ work was unrelated to worries about possible life-and-death consequences of design decisions (participant P20). Participant P02 reported, for example, that working on algorithms for health care “can be really scary” because it’s possible “someone loses their life,” not just that their “Uber didn’t show up.”

Participants pointed to the “hype” surrounding health care MLPA as a driver of potential harms, including claims that current health care MLPA was further advanced than it is or suggestions that its integration into health care is inevitable. Developers described this hype as perpetuating an overly optimist perception of the readiness of health care MLPA for implementation (participants P16 and P35).

### Developer Responses to Drivers of Potential Harms of MLPA in Health Care

#### Overview

Developers described ways to respond to or constrain drivers of potential harms of MLPA, including both opportunities for individuals and organizations to integrate responses into the development process, as well as perceived limitations on developers’ responsibilities to respond to drivers of harms. Opportunities included balancing algorithm performance goals with potential harms; emphasizing the ongoing, iterative integration of health care expertise in the development process; and fostering shared company values. Perceived limitations of developer responsibilities included statements in which developers indicated that the potential benefits of MLPA in health care justified the risk of potential harms of their products; examples of respondents shifting the responsibility for mitigating harms to the end users of this technology and participants’ suggestions that it was the role of regulation specifically to address such risks, even when their knowledge of the relevant policies was limited. Multimedia Appendix 5 provides the longer passages from which quotes cited here are excerpted.

#### Developer Opportunities to Respond to Drivers of Harms

In their discussion of response to drivers, developers identified a number of concrete opportunities within the development process. Regarding balancing performance and risk of harms, participant P16 explained, for example, that their team tried to make their work “very transparent” even though this might require “sacrificing performance” as a singular goal. In doing so, developers could create something that colleagues could examine, which was worthwhile because it allowed “others to be able to look at this thing,” which facilitated a “kind of quality check” by outsiders. Participant P24 explicitly recognized the developer’s role in fostering design decisions that prioritized minimizing potential downstream harms to patients.

In describing the ongoing or iterative integration of expertise as a response to potential drivers of harms, developers echoed the importance of interdisciplinary teams to ensure the presence of clinical expertise throughout MLPA development. An iterative process that engaged interdisciplinary colleagues also allowed developers to ensure that “we’re actually making the predictions...the impact we want to be making,” (participant P14) while at the same time allowing them to “see how the tool interacts with the clinician” (participant P09).

Participants also cited the contribution that shared values among colleagues or at an organizational level could make by creating a context favorable to responding to potential harms and drivers of harms (participant P01). For example, participant P25 described how a “self-aware group of people that are comfortable with humility and vulnerability” could think “through the unintended consequences of a model” by posing questions such as, “how would I feel if I were someone predicted in this model...what would I want to be done with that information?”

#### Developer Limitations in Responding to Drivers of Harms

While some developers were attuned to opportunities to integrate responses to drivers in their everyday work, others focused instead (or in addition) on the limitations of developers’ roles of responsibilities to do so. One way in which participants emphasized the limitations of their role was by suggesting that the benefits of their work could justify the risks of harm. For example, while participant P15 recognized “all sorts of really terrible uses of machine learning,” they then followed this statement by balancing this concern with the desire to see “machine learning helping medicine.” Participant P20 provided a more detailed account of perceived benefits, arguing that although there may be potential harms to patients, ultimately their work is “all about being able to make sure that as many as people possible have health care benefits.”

In addition to positioning potential benefits as a justification for potential harms of MLPA, developers’ statements regarding the limitations of their own responsibilities pointed to the roles of others—and particularly the end users—in preventing (or perpetuating) these harms (eg, participant P31). In particular, developers emphasized the role of the clinician as a safeguard against potential harms to individual patients (eg, participants P09 and P40) and suggested it would be the clinician’s responsibility to understand the details of any algorithms used in order to apply results appropriately (participant P14).

Finally, some developers also recognized a potential role for regulation as a tool for responding to drivers of MLPA-associated harms, including by protecting patient privacy (participant P38), and as a mechanism for increasing public trust in the technology and thus advancing its acceptance (participant P13). However, overall, developers did not evidence a high level of awareness of laws and regulations pertaining to MLPA applications in health care (participants P37 and P07) and expressed skepticism regarding the effectiveness of regulation due to the iterative nature of their products (participant P16).

## Discussion

### Principal Findings

Our findings suggest that, as a group, MLPA developers working in varied roles and organizational settings are able to identify a number of potential harms of MLPA in health care previously noted in the literature, including risks to privacy and bias, among other concerns [41-44]. Some developers also illustrated a more nuanced understanding of these issues through their ability to identify drivers of potential harms. Specifically, developers recognized ways in which the application of MLPA in the health care setting raised the additional potential for harm, both because of the sensitivity and complexity of health care data and delivery, and because of the increased stakes of predictions made by MLPA models. Moreover, as MLPA operates at the intersection of health care and the high-tech industry, “hype” was identified as a driver of potential harms, which commentators have tied to the pressure associated with the commercialization and translation of other types of biomedical products [45]. Of particular note, participants recognized not only potential harms affecting individual patients, but also those that would impact clinicians and the broader health care system. This suggests that at least some developers’ ability to identify ethical issues was not limited to the individual-level impacts that are the focus of much of the literature [44]. Further, developers cited responses to potential harms directly at their, or their organization’s, disposal, including efforts to improve the transparency of algorithms and the benefit of shared values across organizational levels, a finding that confirms scholarly attention to organizational context [46,47].

MLPA guardrails will remain partial to the extent that their routine implementation will rely on developers and others to use, assess, and fine-tune, an awareness that motivates some advocates to endorse other MLPA oversight strategies. These include strategies to influence conduct that could complement technical solutions, which are possibly more readily available, and are directed toward individuals who create MLPA, the ML developers. For example, computing organizations, such as GO FAIR (supporting Findable, Accessible, Interoperable, and Reusable data), FairML, Open AI, and the Partnership on AI, articulate and disseminate proposals for an ethically and socially aware worldview for developers [15,48,49]. Along similar lines, organizations such as the Association for Computing Machinery and Organization for Economic Cooperation and Development have developed codes of ethics to “inspire and guide the ethical conduct” among computing professionals [50-54].

The extent that such developer-focused MLPA efforts might impact conduct in ways that reduce concerns about potential MLPA harms, however, remains unclear. Effects of organizations such as GO FAIR or FairML are difficult to evaluate and, in any event, are likely to remain limited to developers aware of these programs, a small group relative to the large numbers of people involved in ML development. The effects of codes of ethics on conduct are less difficult to assess, but the results are not encouraging [55]. Of the recent studies conducted about codes of ethics in computer science and engineering, the positive effects of the ethics codes on conduct are rare [13,17,24,56]. Research examining the Association for Computing Machinery’s code reported that having participants consider the code when making design decisions had “no observed effect” when compared with a control group [13].

In light of the broad interest in ethics codes to help manage conduct [55,57], it is important to consider possible reasons for their limited success. Some research has framed the question as individually based, for example, asking developers to apply a code’s principles to particular problems [13,55]. Other research recently has taken issue with this framing and has turned attention instead to the role that organizations play in communicating and supporting the principles that codes endorse. Heger et al [14], identify a principles-to-practice-gap in the integration of principles into daily work decisions and conclude that to bridge the gap, organizations must develop policies and activities that align ethics-related activities across 4 levels within an organization: individuals, teams, organizational incentives, and mission statements. Additional studies push for more attention to organizational context [46,47], while the study by de Ágreda [58], which compared acceptance of ethical codes designed to govern development of AI technologies for use in military versus nonmilitary settings, concluded that an important characteristic for success of transferability of codes across settings is the way individuals understand the work an algorithm-driven tool is meant to accomplish.

This idea is echoed in other studies cited here and suggests that future research might benefit the field by focusing on ML developers [17,24,56,59]. Unlike prior interest in individuals, directed toward assessing their knowledge of an ethics code’s content [13], these proposals draw attention to the possible benefit of examining developers’ understanding of the relevance of ethics to their daily work. For example, a recent bibliometric review of research in codes of ethics, encompassing over 100 studies, drew attention to 1 set of studies as demonstrating that ethical breaches might persist in the workplace because employees simply do not connect the code with their work [56]. Similarly, a critical analysis of the current state of AI ethics literature concludes that problems might persist in this domain because the workers themselves are unclear about potential problems their work creates [17]. Possibly lending support to this theory, Mittelstadt [25] points out that unlike medicine, in which practitioners are trained to be highly concerned with protecting patients’ interests, AI development prioritizes commercial success, resulting possibly in deflecting attention away from problems associated with implementing AI tools.

Research that examines ideas about responsibility in AI also underscores interest in examining ML developers’ understanding and attitudes. The few relevant empirical studies to date suggest that developers may be unaware of the harms their work might generate [17] and that developers largely view their responsibility in responding to these potential harms as limited to solving technical problems [60]. Reasonably, much of this scholarship takes the concept of “responsible AI” as its starting point [47,61,62]. Some, however, fail to distinguish responsibility in AI from the formal concept, “responsible AI.” The point here is not that “responsible AI” is not responsible. Rather the point is that the meaning and practices constituting responsibility in AI can extend beyond the highly formalized concept of “responsible AI.”

Understanding this, for example, allows Widder and Nafus [62] to problematize “responsible AI” and to investigate whether and under what conditions the framework solves, or fails to solve, and the problems it is meant to address. This greater latitude directs attention away from higher-order concepts, such as ethics codes or principles, if only temporarily, and toward investigating foundational issues that influence the effectiveness of concepts such as “responsible AI,” for example, how developers understand their work or its potential for harm. Our research findings provide valuable empirical insights to inform our understanding of the persistent principles-to-practice gap, in particular regarding developers’ lack of clarity concerning responsibility for handling various types of issues, a result that confirms the findings of Widder and Nafus [62] about the weaknesses of formal paradigms, such as “responsible AI,” to effect change.

The range of harms, drivers, and potential responses to these drivers identified by developers in our study suggest the basis for a set of domains and preliminary conceptual framework for a broader evaluation of developer understanding of the complex, multidimensional impacts of implementation of MLPA in health care. The extent to which the depth of developer knowledge and recognition of these issues varied within our sample suggests that the need for interventions to systematically educate developers about potential harms and the role of development teams in mitigating harms at all stages of the design process, remains necessary. Systematic measures to evaluate developer understanding of these issues will be essential for evaluating any such intervention strategies designed to bridge the principles-to-practice gap. Future research also may build on our findings to further investigate individual and organizational correlates for greater understanding of these multidimensional challenges among developers.

Perhaps most importantly, our findings suggest that assessment of developer knowledge and understanding of the potential harms of MLPA in health care and their drivers should also include an assessment of individuals’ perceptions of their own roles and responsibilities in responding to these drivers and mitigating against potential harms of MLPA in health care. Tackling the principles-to-practice gap ultimately will require not only systematic developer education, but also a nuanced empirical understanding of the concrete steps developers can take together with a normative understanding of their ethical obligations, to integrate these considerations in their daily work.

### Limitations

This study represents a qualitative analysis of the perspectives of MLPA developers regarding potential harms and regulation of their products and, thus, was not designed to address questions of frequency or to be broadly generalizable. While in the aggregate, participants recognized a range of harms and aspects of regulation that could address these harms, our findings regarding harms, drivers, or responses to drivers were not reflected in statements by every respondent. Although we were not able to identify any individual or organizational characteristics associated with a greater understanding of the issues articulated above, it is possible that our sample was too small to identify respondent characteristics associated with particular types of responses or perspectives. Examination of the role of such characteristics on attitudes toward accountability and responsibility will require further research. Furthermore, MLPA developers not included on LinkedIn would only be reached through snowball sampling, and our sample was drawn primarily from private sector organizations, where MLPA currently implemented in health care in the United States is largely being developed [36], so we do not know whether our findings are generalizable to MLPA developers in other settings.

### Conclusions

Our findings suggest that developers within the setting of commercial development of health care MLPA in the United States recognize a range of potential harms of MLPA to individuals, groups, and health systems. They also can articulate various drivers of these harms located in the characteristics of MLPA itself, those of the health care and commercial environments in which they are implemented, as well as drivers stemming specifically from the intersection of these domains. While some developers also indicated recognition of their responsibility to respond to some harms, others displayed more limited views. While broad education of MLPA developers about the potential harms of their products may be necessary, it will not be sufficient if developers do not recognize or accept a role in mitigating those harms. Furthermore, measures to address the challenges posed specifically by the health care and business contexts of MLPA development may be necessary to minimize harms.

## Appendix

Multimedia Appendix 1 Interview guide.

Multimedia Appendix 2 Individual participants’ academic backgrounds, data interaction levels, and management levels, organized numerically by participant ID (n=40; data for participants P04 and P27 were incomplete and so were excluded from analysis).

Multimedia Appendix 3 Potential harms of machine learning predictive analytics (MLPA) in health care.

Multimedia Appendix 4 Drivers of potential harms of machine learning predictive analytics (MLPA) in health care.

Multimedia Appendix 5 Developer responses to drivers of potential harms.

## Abbreviations

AI: artificial intelligence
FDA: Food and Drug Administration
ML: machine learning
MLPA: machine learning predictive analytics
SaMD: software as a medical device

## Data Availability

The deidentified data sets analyzed during this study are available from the corresponding author on reasonable request.
