# Supplementary material for: Developer Perspectives on Potential Harms of Machine Learning Predictive Analytics in Health Care: Qualitative Analysis
Source: J Med Internet Res. 2023 Nov 16;25:e47609. doi: 10.2196/47609 (PMC10690528; doi:10.2196/47609)
Supplement: Multimedia Appendix 1 [file jmir_v25i1e47609_app1.docx]

Part 1: Background: training, roles, and project

1. Can you tell me about your background, training and education?
2. How long have you been working with this [group/company]?
3. Can you tell me a bit about the specific ML project, or projects that you are involved with?
4. How will this product be used?
   1. Possible probes: in what settings, by whom, for what purpose, to achieve what goals?
      1. NOTE: If R does NOT frame product/program’s purpose or goals as predicting something, ask:
         1. Does the project include a goal <<to improve prediction of>> OR <<to predict>> [fill-in phrase that describes project/program]?
5. What is/was your role on this project?
   1. Possible probes: developed a particular feature, machine learning expertise, clinical research expertise, providing access to data sources, clinician or patient perspectives, project management, oversight etc.
      1. Establish whether R contributes multiple skills/tasks?

Part 2: Domain Expertise [Choose 1 or 2]

1. If R IS the team member with health-related expertise, refer back to R’s domain expertise based on Part 1 answers or other information provided, and ask:
   1. Can you tell me about the extent to which you were involved in the project?
   2. Can you tell me about some examples of how you contributed your expertise?
   3. Depending on response, ask
      1. <<Are your suggestions usually unprompted>>, OR <<Are your suggestions usually in response to a query by team members? >>
      2. Did the team apply your suggestions? [Try to get at possible disjuncture between what R suggested and what team thought was needed
      3. Have R’s teams’ needs ever exceeded R’s expertise?
         1. If yes, how handled? [Possible prompts: is there anything you do to keep your expertise current? Does your company support/recognize your need to do this
2. If R is NOT the person with domain expertise, refer back to Part 1 answers and ask, was/is there a team member
   1. What kinds of expertise are needed on your team?
   2. On this project, whose role was to provide the expertise?
   3. Did you feel you needed health-related expertise?
      1. What kind of expertise was it?
      2. How did you get it? In-house or external? What was the nature of their involvement on the project?
      3. Can you give me some examples of the kinds of questions would you ask the person with health-related expertise?
   4. Did the answer allow you/other team member to move forward?

Please elaborate.

Part 3: Barriers, Facilitators, and Challenges

1. Reflecting over your experience <<or over recent projects>>, can you discuss [OR give some examples of] how teams handle setbacks in their work?
   1. Can you elaborate? [Possible follow-ups re a specific example:]
      1. What trade-offs did the teams have to make?
      2. Did you solicit help outside the immediate team?
      3. Was time a factor – If so, how was that conveyed to you?
      4. Was the outcome satisfactory?
2. [IF additional detail re: Barriers and Facilitators NEEDED, e.g., re trade-offs or outreach beyond the immediate team, etc., try asking:
3. If you were to get a do-over for this project, what one or two things might you want to change or do differently? For example: [choose among the following probes:]
   1. Questions you wished you had asked?
   2. Other people or expertise you wish you had included? Or would definitely include again?
   3. Additional resources you wished you had had?

Part 4: Unintended Consequences

1. Who do you think might benefit the most from the product? How?
2. Do you think there might be some unintended consequences of this program when implemented?
   1. Please elaborate.
   2. Did the team ever discuss such possibilities? For example, Was there any discussion of how they could be handled differently? Please elaborate.

Part 5: Regulation

1. Do you think federal regulation of these types of products is called for?
   1. Why or why not?
2. As you may or may not be aware, the FDA has proposed regulating healthcare AI [such as R’s product] based on what they are calling, “a culture of excellence” in AI development. As opposed to a product-focused regulatory framework as is used for pharmaceuticals, this regulatory framework focuses on examining the AI development process itself and the “organizational culture” of the company/developers.
   1. Have you heard about these proposals?
   2. [*If no*] What would a culture of excellence in AI development mean to you? Do you think they could work?
   3. [*If yes*] What do you think about these proposals? Do you think they could work?
      1. For both: Can you elaborate on your response?

Part 6: Closing

1. Are there other questions I should ask?
2. Do you have questions for me?
3. Are there other people I should talk to?
