# Supplementary material for: Developer Perspectives on Potential Harms of Machine Learning Predictive Analytics in Health Care: Qualitative Analysis
Source: J Med Internet Res. 2023 Nov 16;25:e47609. doi: 10.2196/47609 (PMC10690528; doi:10.2196/47609)
Supplement: Multimedia Appendix 2 [file jmir_v25i1e47609_app2.docx]

| **Participant ID** | **Academic Backgrounds** | **Data Interaction Levels** | **Management Levels** |
| --- | --- | --- | --- |
| P01 | Non-health-related PhD | Data + | High-level |
| P02 | MD | Data Only | Mid-level |
| P03 | Bachelors | Data Only | High-level |
| P05 | Bachelors | Data + | High-level |
| P06 | Non-health-related Masters | Data Only | High-level |
| P07 | Non-health-related Masters | Data Only | None |
| P08 | Bachelors | Data + | Mid-level |
| P09 | Non-health-related Masters | Data Only | Mid-level |
| P10 | Bachelors | Data Only | High-level |
| P11 | Health-related Masters | Data + | Mid-level |
| P12 | Health-related PhD | Data + | None |
| P13 | Bachelors | Data + | None |
| P14 | Health-related PhD | Data Only | High-level |
| P15 | Health-related PhD | Data + | High-level |
| P16 | Health-related PhD | Data + | High-level |
| P17 | Health-related PhD | Data Only | Mid-level |
| P18 | MD | Data + | None |
| P19 | MD | Data + | Mid-level |
| P20 | Non-health-related PhD | Data + | High-level |
| P21 | MD | Data Only | Mid-level |
| P22 | Non-health-related PhD | Data Only | High-level |
| P23 | Non-health-related Masters | Data + | None |
| P24 | Non-health-related PhD | Data Only | High-level |
| P25 | Health-related Masters | Data Only | Mid-level |
| P26 | Non-health-related Masters | Data + | None |
| P28 | Bachelors | Data + | Mid-level |
| P29 | Non-health-related Masters | Data + | None |
| P30 | Bachelors | Data + | None |
| P31 | Non-health-related Masters | Data + | None |
| P32 | Health-related Masters | Data + | None |
| P33 | Bachelors | Data Only | High-level |
| P34 | Non-health-related PhD | Data + | None |
| P35 | Non-health-related PhD | Data Only | High-level |
| P36 | Bachelors | Data + | None |
| P37 | Non-health-related PhD | Data + | None |
| P38 | Health-related Masters | Data + | None |
| P39 | Health-related Masters | Data + | None |
| P40 | Bachelors | Data + | High-level |
| P41 | Health-related PhD | Data Only | High-level |
| P42 | Non-health-related PhD | Data + | High-level |
