# Supplementary material for: Developer Perspectives on Potential Harms of Machine Learning Predictive Analytics in Health Care: Qualitative Analysis
Source: J Med Internet Res. 2023 Nov 16;25:e47609. doi: 10.2196/47609 (PMC10690528; doi:10.2196/47609)
Supplement: Multimedia Appendix 3 [file jmir_v25i1e47609_app3.docx]

| **Individual Harms** | |
| --- | --- |
| *Misdirection of Health Care* | **P02:** “I worry a lot about, you know, if the placement of people in buckets, even if you do segment people, deciding, you know, how many segments to have and exactly where those cutoffs are is a real problem.”  **P24**: “… ‘cause it’s simply just math and numbers…it’s hard to realize that hey, somebody could actually not get treatment or a claim for somebody could be denied because you built a claims adjudicator algorithm…you can fine tune your algorithm to be let’s say more precise or be more specific…and both have different implications. Maybe a false-positive - like paying a claim that shouldn’t have been paid – has less implications than denying a claim that should have been paid… I think I would rather that people have their claims paid than denied, so I will just tune it for true-positives. Obviously, you have to be within a certain boundary.” |
| *Privacy Violations* | **P30**: “The patients don’t know that there’s a bunch of people looking at their PHI… and we were looking at everything, social security numbers would even come up. Sometimes counselor visits would show up and you can see private conversations that are happening between a counselor and a patient, but I mean that was pretty rare, but it did slip in there sometimes.” |
| **Group Harms** | |
| *Impact on Sociopolitical Groups* | **P06: “**There was a study published in Nature Journal this year, which was racial biases in population health algorithms...And that was a fantastic and eye-opening study for a lot of people, but, you know, I think that that is exactly the, you know, the types of issues you think about… those algorithms are used by all health insurers, all big provider groups, you know, 150 million lives go through those algorithms and, you know, it underscores African-Americans in a pretty significant way…”  **P17**: “if you’re truly optimizing based on cost you can get like… you know, they have these like racially biased algorithms that don’t take into account all things so they improperly assign risk to people and not controlling for other factors like socioeconomic status and then like geolocation for instance.” |
| **Health Care System Harms** | |
| *System Disruption* | **P19:** “Because, you know, if the algorithm can do a lot more of the grunt work in terms of figuring something out, if it can do the pattern recognition stuff as well as the clinician does, and you know certain skills tend to atrophy over time.”  **P08:** “So alarm fatigue on one side and on the opposite side, if clinicians become sort of overly reliant on our tools that may systematically affect their judgment in the opposite direction.”  **P22**: “So if you deploy a machine learning algorithm and you can forget about it and then press the retrain button three years later… you may find yourself in a position where the machine is sure that you should not take the vaccine anymore because, you know, how does the machine know that the vaccine… is useful… And just like in the antivax movement, if there’s no examples of kids with mumps or rubella or whatever, right, then you forget the causal relationship between the two and then you’re… you know, junk comes out. So my fear is that, you know, as the systems get deployed, if we’re not really careful we may be setting ourselves up for later down the road having systems that aren’t… gonna respond to the data that’s generated by, you know, the backload of data that’s examined will not contain the relevant information any more. So how do you battle that? You have to be careful when you design the system. So this is kind of one of my like scenarios where, you know, that the machine becomes the antivax.” |
| *Wasted Resources* | **P20:** “So there’s also, in my mind, a concern that well we could see this wave of machine learning health care companies that focus on the types of problems that you can solve with machine learning, which is a good thing, but if that crowds out the other kinds of investments that could be made, then maybe not as good of a thing.”  **P22:** “But machine learning is not infrastructure because, you know, if you build a fancy machine learning model and you don’t get, you know… and you spend a ton of money for it and then no one uses it, it’s wasted.” |
