# Supplementary material for: Developer Perspectives on Potential Harms of Machine Learning Predictive Analytics in Health Care: Qualitative Analysis
Source: J Med Internet Res. 2023 Nov 16;25:e47609. doi: 10.2196/47609 (PMC10690528; doi:10.2196/47609)
Supplement: Multimedia Appendix 4 [file jmir_v25i1e47609_app4.docx]

| **Factors Developers Characterized as Inherent in MLPA Technology** | |
| --- | --- |
| *Opacity* | **P18**: “… to me that’s the scariest thing about machine learning in general, is if you have a model that’s not really explainable and it’s pretty predictive and you don’t know why it’s predictive, that’s actually a recipe for [laughter], you know, a disaster. Because, you know, what if it’s… what if it’s actually using some surrogate variable that you don’t want it to predict?”  **P35**: “…it [an MLPA algorithm) incorporates the data on which it’s trained, and if that dataset includes things like racial bias, then you are, you know, solidifying racial bias in a tool in a way which is opaque to the people who are using it.” |
| *Limited Generalizability* | **P19**: "…in the machine learning world… most of the papers actually you will see [are] applied to a very narrow subset of patients in a very specific set of circumstances. I mean my patient has a cold on a full moon night, what does this model tell me about how he’s gonna be? And this doesn’t really generalize well. The idea that your model has taken the shape of whatever data you’ve seen… actually shown to it… Sheltered existence if you will.”  **P08**: “It is a significant issue in machine learning, as in the entire field of study, that the metrics that we use are one, either ill-equipped for what we actually mean, or are falsely representative of performing seeing real life. So you can often see claims made by machine learning companies that their software is 99% accurate…When in the actual use case they are more like 80% accurate because of institutional idiosyncrasies between different contexts.” |
| *Data Volume* | **P03**: “You can screw up just as badly with a logistical regression as you could with a… which is kind of a form of AI …as with a neural net. You can just make much bigger mistakes with a neural net…as you can look at bigger data, but if you… if you start with, you know, garbage in equals garbage out, no matter where you’ve… where you’re playing.”  **P20**: “You know, a lot of people who train on AI/machine learning, that sort of thing, they use genetic datasets or they use imaging datasets, right, ‘cause that’s where you can get large volumes of data and a lot of the people who go into those fields are coming from CS backgrounds too, so they… the more data, the better, right, like it’s like exciting. And at the end of the day it’s… what you learn from dealing with a genetics dataset or an imaging dataset just doesn’t translate at all to health care clinical data or claims data for that matter.” |
| **Health Care Environment Characteristics** | |
| *Structure of Health Care Data* | **P24**:” So a lot of the limitations come from your data itself, right, and data… well maybe the way I would classify it is a lot of limitations come from infrastructure itself, like data infrastructure, and then a lot of limitations come from processes as well, so just the way a claim is submitted, like just the way an EMR is kind of stored and sent, right, like this lack of common protocols**.”**  **P42**: “…health care data is really messy too…What do I mean by messiness? The fidelity of the data, the accuracy with which it is entered, the timeliness with which it is entered …the formats with which it is entered, the way in which that can get scrambled or screwed up because there’s a bunch of different EMRs… and you get into this really easily garbage in, garbage out scenario. The problem is you don’t realize that it’s garbage in. You get a dataset and you think all right, great, I’m gonna build this fantastic model, and you don’t realize that you’ve just overfitted a model to a big pile of garbage and that sounds really kind of harsh.” |
| *Sensitive Information* | **P30:** “but the one thing that… and maybe this ties in with like medical ethics, is that the patients don’t know that there’s a bunch of people looking at their PHI and yeah… so, and we were looking at everything, like social security numbers would even come up. Sometimes counselor visits would show up and you can see like private conversations that are happening between a counselor and a patient, but I mean that was pretty rare, but it did slip in there sometimes.”  **P05**: “It wasn’t anything sinister, but… the clinical team was brought in to make sure that, you know this sort of approach was appropriate… more of a team effort to have those conversations when we were doing a little bit more clinically or socially more sensitive content.” |
| *Complexities of Health Care Delivery* | **P33**: “Another thing could be understanding the fact that, you know, sometimes people will do training datasets on CAT scans or MRIs or whatever… not realizing that there are nuanced difference between different types of machines and so you may build a model off of your GE thing and it’s useless on your Siemens thing.”  **P05**: “…especially with, you know, harder to understand conditions or, you know, especially cancer staging… when you get into the really nuanced side of medicine where it’s, you know, chemotherapy regimens and what’s appropriate, what’s not, and especially interpreting new offerings on these things, it very quickly just gets into very murky waters.” |
| **Intersection of Health Care and the High-Tech Industry** | |
| *Disparate Perceived Responsibilities for Addressing Risk* | **P03**: “I think there are some issues there, but I think the bigger... the disconnect that I’ve seen is in the ethos of how ... computer science has turned into a business... built around this notion of tolerance to failure. The cost of doing it is cheap, so we can fail and move on, do another one. That is antithetical to medicine. You’re not allowed to fail with people”  **P20**: “the stakes are pretty low when Google’s imaging detector like calls a cat a dog, right…but if you’re gonna use that same kind of imaging technology to power automated driving… self-driving cars, then… it needs to be tested and tested and tested. I think that’s the same kind of thing that needs to happen in health care. The only thing is I don’t know what the low stakes thing in health care is, right.”  **P02**: “One thing to ask [about an algorithm] is, and it can be really scary to ask, is but like kind of the worst-case scenario and I think that’s a lot of the problem with health care is, you know, the worst case is that someone loses their life, you know, I mean or gets worse even, it’s just... versus, you know, someone’s Uber didn’t show up. It’s a different... it’s a different worst case...” |
| *Hype* | **P16**: “I think there’s like a bit of a disconnect between how machine learning in health care is presented in the media and the sense that the public has about how much data we really have and the amount of actual tangible data that a given health system, even a big one like [P16.’s health system organization], actually has available to build these models.”  **P35**: “All of a sudden everything has AI in it. You know, you turn around, you know, and it’s like your toothbrush has AI in it or whatever. And it’s sort of a skinned knee and you sort of dust off the front of it and there’s like nothing there….It’s almost like completely devoid of any real meaning. Not to say there aren’t people that have done meaningful things in AI and I think the signal processing is one area that there have been important advances, which may change things…I think there’s some real value in that stuff, but by and large it is… it’s all sizzle without substance.” |
