# Supplementary material for: Developer Perspectives on Potential Harms of Machine Learning Predictive Analytics in Health Care: Qualitative Analysis
Source: J Med Internet Res. 2023 Nov 16;25:e47609. doi: 10.2196/47609 (PMC10690528; doi:10.2196/47609)
Supplement: Multimedia Appendix 5 [file jmir_v25i1e47609_app5.docx]

| **Developer Roles and Responsibilities** | |
| --- | --- |
| *Balancing Performance and Potential Harms* | **P16**: “There are teams that are doing very … I don’t know what the right word is… modern machine learning, where it’s like just throw data in there, get an outcome. In our case, we’re using machine learning, but in a way that makes the workings of the model very transparent so we can kind of quality check it, like an outsider could look at it and eyeball it much more easily. And we did that even knowing that we might be sacrificing some performance because we felt it was important for like [Colleague’s name] and others to be able to look at this thing.”  **P24**: Now, a data scientist has tremendous powers here because like your stakeholders don’t really understand what precision recall is and where that threshold should be, so it’s up to you to use your own judgment and say, you know what, actually I think I would rather that people have their claims paid than denied, so I will just tune it for true-positives. |
| *Iterative Integration of Expertise* | **P41**: “We’ll build the model, then after … we’ve got a first pass, we’ll send them, you know, example people that we’ve highly prioritized by the model and we’ll ask them to like go through and say okay, does this make sense, like are we actually finding people that would be useful for this situation and… yeah, we need their feedback to iterate and see if like we’re actually making the predictions… the impact we want to be making.”  **P09**: “…[I]deally it [defining a research problem] would be an iterative sort of back and forth process where, you know… it would be engineers and clinicians working side by side the whole time together and really getting to see how the tool interacts with the clinician.**”** |
| *Shared Values* | **P01:** “…what I want to see [P01’s company] do is be the moral leader and hopefully, you know, they’ll go, hey look, we can make a quick buck this next quarter, but let’s do the right thing and let’s set the standard instead of taking advantage of the fact that the regulatory landscape right now is the wild, wild west…”  **P25**: “but if you really want to think about these models being used in the right way, you have to have a self-aware group of people that are comfortable with humility and vulnerability. Because that’s also part of thinking through the unintended consequences of a model, right, is being able to think about well how would I feel if I were someone predicted in this model…what would I want to be done with that information?” |
| **Perceived Limits of Developer Roles and Responsibilities** | |
| *Benefits Justify Risks* | **P15**: There’s been all sorts of really terrible uses of machine learning that mostly penalize people that are already penalized in lots of other ways, like people of color or other kind of minorities. It’s just sort of amplifying all these other bad things that are already happening….but I’m also not like a person… you know, I want to be able to do machine learning and have progress and see…machine learning helping medicine, ‘cause it has so much that it can offer I think.  **P20**: There was a data scientist who was concerned…that we were moving away from our mission, like now we’re all about pricing and helping health plans make money, but I put on kind of my company man/economist hat and said …the ACA has had a lot of problems because of mispricing and that in a lot of counties only one plan is able to operate because there’s just too much risk to price there… it’s all about being able to make sure that as many as people possible have health care benefits. But if you misprice things dramatically and you have adverse enough selection, then that doesn’t benefit everybody. |
| *Shifting Responsibility to the End Users* | **P31**: I mean it depends on how the analytics is used and the purpose and the motives and the intention of the users. But as producers of analytics, we intend them to be used for general good.  **P09:** It totally leaves it in the clinician’s hands. The clinician understands the context within which the prediction is made and they know that, you know, it’s up to them to decide whether or not the patient should be treated. It’s really just an indicator.  **P40**: With a recommendation you have a clinically trained person who’s supposed to use and synthesize that information along with everything else they’ve been taught and there’s a point of manual… there’s someone thinking about this before it happens.  **P14**: If I’m a doctor let’s say using this algorithm and I see that it was trained on a patient panel that looks nothing like mine, I’m probably not gonna believe the results, you know, and I think for a good reason. |
| *(Over)Reliance on Law and Regulation* | **P38**: “I believe there should be [regulation] because the data that we handle is like very, very sensitive, so there should be regulations.”  **P13**: "But in terms of building trust, I think a bunch of certifications definitely can help. Like for one we got… last year we had our HITRUST certification and so that was definitely something that got us a few deals from some big companies, which otherwise wouldn’t have happened.”  **P37**: …well I guess first of all I think there’s enough of a legal structure in place that it’s like you can’t… that insurance plans can’t drop people for… because they think they’re gonna be high cost in the future.  **P07**: “First of all I don’t know why FDA would be involved in claim data health care stuff, but it’s not related to food and drugs”  **P16**: “So I think it’s really hard, if you talk about regulating a specific AI product, does that mean that every time you retrain the model on a new dataset you have to go back to the FDA and get them to approve it again? Does that mean if you think of a way to improve the model you have to go back and get their approval for that? That seems like it would just be impossible to do when you’re developing multiple different models at once.” |
